# Extractable impurities from fluoropolymer-based membrane filters – interference in high-throughput, untargeted analysis†

**DOI:** 10.1039/c9ra06198c

**Published:** 2019-10-08

**Authors:** Perng Yang Puah, Dexter Jiunn Herng Lee, Ken Hing Mak, Hui Jun Ang, Hsing-Chang Chen, Pak Yan Moh, Siat Yee Fong, Yee Soon Ling

**Affiliations:** Faculty of Medicine and Health Sciences, Universiti Malaysia Sabah Jalan UMS 88400 Kota Kinabalu Sabah Malaysia siatyee@ums.edu.my; Biotechnology Research Institute, Universiti Malaysia Sabah Jalan UMS 88400 Kota Kinabalu Sabah Malaysia; Faculty of Sustainable Agriculture, Universiti Malaysia Sabah Locked Bag No. 3 90509 Sandakan Sabah Malaysia; Institute of Food Safety and Health, National Taiwan University No. 17, Xu-Zhou Rd Taipei Taiwan 10055; Faculty of Sciences and Natural Resources, Universiti Malaysia Sabah Jalan UMS 88400 Kota Kinabalu Sabah Malaysia; Water Research Unit, Universiti Malaysia Sabah Jalan UMS 88400 Kota Kinabalu Sabah Malaysia ling82ys@gmail.com

## Abstract

The removal of particles using fluoropolymer-based membrane filters is usually done so to prolong the life span of an analytical column, prevent hardware damage, and reduce signal suppression. Ironically, these membrane filters tend to leach impurities into the samples as the samples are filtered through them. These impurities have the potential to affect the researcher's interpretation in high-throughput, non-targeted analysis. In this study, extractable impurities from different brands of fluoropolymer-based membrane filters present in the filtrate filtered using the said filters were investigated. The results demonstrated that different brand membrane filters and materials tend to elute vastly different numbers of impurities. There were instances whereby the extractable impurities persisted in both the membrane filter and the filtrate despite the filter being pre-conditioned (up to 3 times). Principle component analysis revealed that filtrates at different purge intervals are distant from the unfiltered samples. Pre-conditioning of the PTFE membrane filters could potentially reduce the number of extractable impurities across the tested brands. PVDF filtrates, however, tend to co-cluster with their respective brands, thus suggesting that dissimilarity persists in brands following conditioning. As such, pre-conditioning of the PTFE membrane filters should be encouraged so as to reduce false positive results, while the use of PVDF membrane filters for mass-spectrometry-based untargeted analysis is not advisable as extractable impurities would still persist after 3 rounds of conditioning. Neither the use of different filter brands, nor the use of different filter materials in a sample batch are encouraged as different membrane materials or brands could potentially elute varying impurities.

## Abstract 2

Investigation of the impurities leaching behavior of the syringe filters.

## Graphical abstract

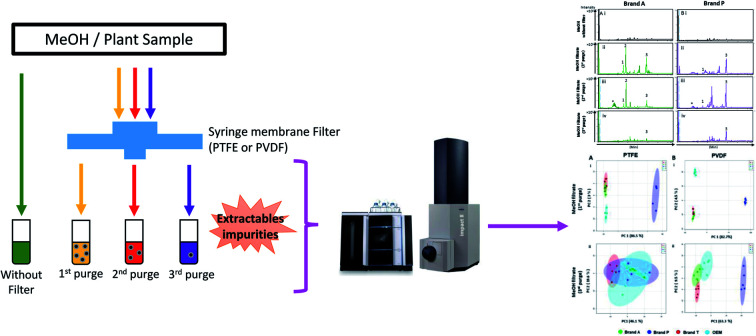

## 1. Introduction

Metabolomics, the most phenotypic-related omics, has been rapidly developing since the late 1990s.^1^ It plays a crucial role as the terminal downstream product of the genome as well as the transcriptome, consisting of the total complement of all the low molecular weight molecules <1500 Da.^2–4^ Its popularity in research and industry was due to the improved mass detectors with advanced sensitivity, accuracy, and compounds (metabolite) mass coverage.^5^ Unlike targeted analysis which aims for accurate, trace level analysis of designated compounds (or metabolite classes ‘known–known’ compounds),^2^ untargeted analysis enables the discovery of ‘unknown–unknown’ and ‘unknown–known’ compounds which can potentially serve as markers throughout biological (diseases), environmental, forensic (toxicology), as well as natural product chemistry.

The availability of ultrasensitive or high-resolution mass spectrometry detectors has enabled researchers to conduct ultra-trace level analysis. The coupling of mass spectrometry with reversed-phased gradient ultra-high performance liquid chromatography equipped with submicron particle stationaries packed column (1.6–1.9 μm) permits the analysis of a broad range of polarity and retention characteristic in a relatively shorter period of time compared to using a reversed-phased isocratic approach. With increased equipment sensitivity and resolution, the presence of ghost peaks in the acquired data is to be expected. Ghost peaks are also known as artifact (artefact) peaks^6^ or pseudo peaks.^7^ The source of these ghost peaks during reversed-phase gradient liquid chromatography from different sources, including mobile phases,^8,9^ improper glassware cleaning (soap residues),^10,11^ and laboratory plasticizers are well documented.^9,12,13^ An elevated background refers to interfering noises that can potentially jeopardize the performance of the mass spectrometer, usually in the form of signal suppression. Additional complications may arise when these unexpected interferences interact with the analytes, thus leading to mass-to-charge ratio shifts that will further obscure spectral interpretations. This commonly results in misinterpretation and wrong compound matching in high-throughput, untargeted analysis.

The presence of particles in prepared samples has the potential to clog liquid chromatographic columns. This will result in the shortening of the said columns' lifespan, particularly those packed with submicron particle stationaries. Apart from column clogs, particle aggregation/precipitation may introduce microscopic scratches on the rotor seal as it turns against the ceramic stator within six-port injection valve. ‘Cross-port scratch’ on the rotor seal can cause sample or eluent leaks during the injection or the analysis phase of the injection. This will result in poor mass transfer, thus resulting in broad chromatographic peaks and poor peak area reproducibility. These particles are also able to negatively affect the signal-to-noise ratio, thus potentially compromising the overall analysis in both absorbance-based and mass-based analyzer. These complications can be negated by physically removing the said particles *via* simple filtration of the prepared samples through a microporous (<0.2 μm pore size) membrane filter.

It is ironic that the very membrane filters that are frequently used to remove particles leach impurities as the samples are filtered through. Previously, emphasis was placed on targeted analysis, whereby specific molecules or compounds were targeted using mass spectrometers such as triple quadrupoles or ion traps. The presence of these extractable filter impurities has minimal impact on targeted analysis in terms of interference. However, emergence of high-throughput, non-targeted profiling onto extractable small molecules, the presence of these extractable filter impurities will affect the interpretation of the acquired data. In this case, it is absolutely important that the devices and products used leaches minimal amounts of impurities (ideally none) into the sample during sample preparation. There are a number of membrane filter materials available in the market, including acetate, nylon, polyethersulfone (PES), polypropylene (PP), polytetrafluoroethylene (PTFE) and polyvinylidene difluoride (PVDF). These membrane materials are packed within PP syringe filters cartridges and are certified as ‘low-extractable’ for applications involving high performance liquid chromatography coupled with absorbance-based detectors, including ultraviolet (UV), visible (VIS) and photodiode array detectors (PDA). These certifications, however, does not apply to mass spectrometry acquisition as mass spectrometers have better sensitivity (low detection limit) and resolution compared to absorbance-based detector.

Among the many organic solvents, alcohols, especially methanol are often used, alone or more usually in mixtures with varying proportions of water.^14^ Being hydrophilic due to their hydrogen-bonding capabilities, methanol is also lipophilic, a property that increases with their chain length; the combination of these features confers a high solubilisation power that allows them to extract a wide range of metabolites. De Vos *et al.* (2007) used acidified water (0.1% formic acid) in 75% methanol (pre-cooled) to extract a wide range of secondary metabolites (flavonoids, phenolic acids, alkaloids and glucosinolates) and reported it to be a very efficient method.^14^ Guo *et al.* suggested that methanol alone able to extract more secondary metabolites of a plant^15^ compared to other solvent mixture. Apart from the extraction efficacy, solvent compatibility with mass spectrometry is another key factor, which needs to be taken into consideration. Methanol (mixture of other organic solvents such as acetonitrile, isopropanol; water, salts and acids as programmed gradient system) is commonly employed as mobile phase to perform complicated chromatographic separation. More importantly, cumulative literature indicated methanol, due to its bipolar properties, is commonly used to reconstitute dried metabolite extracts prior LC or GC mass spectrometry analysis without compromising the sample nor the analytical instrument.^14–17^ Therefore, in this study, methanol is used to demonstrate the impact of filter's extractable impurities on high throughput, untargeted analysis.

Here, we report the investigation of the extractable impurities from fluoropolymer-based membrane filters, particularly PTFE and PVDF membrane filters, as organic solvent (methanol) is passed through these filters. Filtrates were collected and profiled using high-throughput, untargeted profiling. Attempts were made to identify these extractable impurities based on the acquired accurate *m*/*z* and MS/MS signals. An evaluation of the impurities in the filtered plant extracts was carried out.

## 2. Experimental

### 2.1 Preparation of blank methanol in different syringe filter experimental workflow

**Fig. 1:**
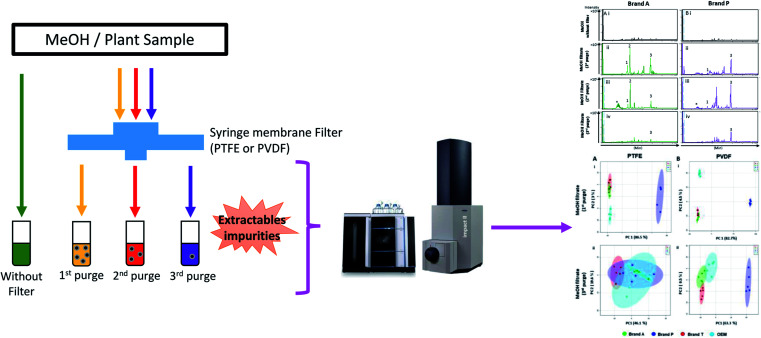
Overall experimental workflow.

The overview of the experimental flow is illustrated as per Fig. 1. Two different types of fluoropolymer-based membrane filter, polytetrafluoroethylene (PTFE) and polyvinylidene fluoride (PVDF) (*n* = 5 for each type), were acquired from different vendors namely Brand A, Brand T, original equipment manufacturer (OEM), and Brand P, with brands A, T, and OEM having a pore size of 0.22 μm and a diameter 13 mm diameter while Brand P has a pore size of 0.2 μm and a diameter of 17 mm. These filters were used to investigate the impurities leaching behavior of syringe filter (including its casing materials) used in the manufacturing of syringe filters.

Briefly, the syringe filters were conditioned (purged) thrice using 1 mL of LC-MS grade methanol (Merck, Darmstadt, Germany). The filtrates from each purge intervals (from the same filter) were collected and subjected to ultra-high performance liquid chromatography (UHPLC) conjugated with both a diode array detector (DAD) and a quadrupole time-of-flight (QTOF) mass spectrometer. Prior to sample (filtrate) injection, a blank (unfiltered LC-MS grade methanol) was injected and analyzed using UHPLC-DAD-QTOF. Profiling was carried out using a diode array detector at various wavelengths followed by a mass spectrometer, with the mass analyzer *m*/*z* range set to 50–1500. Heated electrospray ionization was used and set for positive mode ionization. The acquired data was pre-processed using MZmine 2,^18^ after which chemometric analysis was carried out using Metaboanalyst 4.0 (ref. 19 and 20) and molecular formulae/structure prediction of the detected impurities was done using MS Finder.^21^

### 2.2 UHPLC-DAD-QTOF acquisition

10 μL filtrate was introduced to the Vanquish UHPLC system (Thermo Scientific, Waltham, MA, USA), which was coupled with a DAD (Thermo Scientific, Waltham, MA, USA) followed by ultra-high resolution QTOF, Impact II (Bruker, Billerica, MA, USA), using positive electrospray ionization mode. Chromatographic separation was carried out using a Kinetex F5 column (2.1 × 100 mm × 2.6 μm; Phenomenex, Torrance, California, USA) maintained at 40 °C. The mobile phase used consists of solvent A, which is ultrapure water with 0.1% formic acid and 1% 1 M ammonium acetate (NH_4_AC) added; and solvent B, a mixture of acetonitrile and methanol [6 : 4 v/v] with 0.1% formic acid and 1% 1 M NH_4_AC added. The mobile phase was delivered at a rate of 0.5 mL min^−1^ during the duration of the data acquisition. The gradient used for the mobile phase was set as per the following: 1% to 60% solvent B in a linear increase for 7 minutes, then from 60% to 100% solvent B for the next 4 minutes, followed by a reversal to 1% solvent B for the next 4 minutes. Analyte absorbance chromatograms were acquired *via* a DAD (Thermo Scientific, Waltham, MA, USA) at 190, 254, 270, 360 nm. The mass spectrometer data acquisition was set between 50 to 1500 *m*/*z*. The voltage for the electrospray ionization (ESI) (positive mode) was set as 3.5 kV while the gas temperature of the ion source was set at 300 °C, along with the drying gas flow at 10 L min^−1^, and the nebulizer flow at 3.0 bar. The mass spectrometer was calibrated with Tune Mix (Sigma-Aldrich, St Louis, MO, USA) before batch analysis. The mass calibrant, 10 mM sodium formate (Sigma-Aldrich, St Louis, MO, USA), was introduced between 0.1–0.3 min *via* a 6-port 2 positional valve during each sample acquisition. During the post-acquisition, the acquired *m*/*z* were calibrated against the sodium formate introduced in the beginning of the acquisition.

### 2.3 Chemometric analysis

MZmine 2.0 (ref. 18) was used to pre-process the acquired raw data so as to reduce the variations in retention times and *m*/*z* values between the analyses. The processed data was exported as a Metaboanalyst compliant peak list table in CSV format. The exported peak list table was imported to Metaboanalyst 4.0 (ref. 19 and 20), where the peaks (data sets) were subjected to pre-processing using log-transform and Pareto scale prior to multivariate analysis. All data sets were subjected to principle component analysis (PCA), an unsupervised pattern-recognition technique, to determine the differences between filtrates from different purge counts. The molecular identification of the impurities was carried out using MS Finder.^21,22^

### 2.4 Effects of extractable impurities on analysis

Leaves of *Eleusine indica* were collected from Kota Kinabalu, Sabah and kept frozen (−80 °C) overnight prior to freeze drying (Labconco, Kansas City, MO, USA). The freeze-dried samples were homogenized. Extraction was done on 10 mg of the homogenized sample using a mixture of chloroform, methanol and double distilled water (1 : 1 : 1, v/v/v).^23^ The non-polar layer (lower partition) was collected from separatory funnel and vacuum concentrated so as to produce a semisolid crude extract. This semisolid extract was re-suspended in LC-MS graded methanol and centrifuged at 6000*g* for 20 minutes. The resulting supernatant was equally divided into multiple parts, with each part being filtered once using either unconditioned PTFE, unconditioned PVDF, pre-conditioned (purged 3 times) PTFE, or pre-conditioned PVDF membrane filter. The resulting filtrates and supernatant were profiled using UHPLC-QTOF.

## 3. Results and discussion

**Fig. 2:**
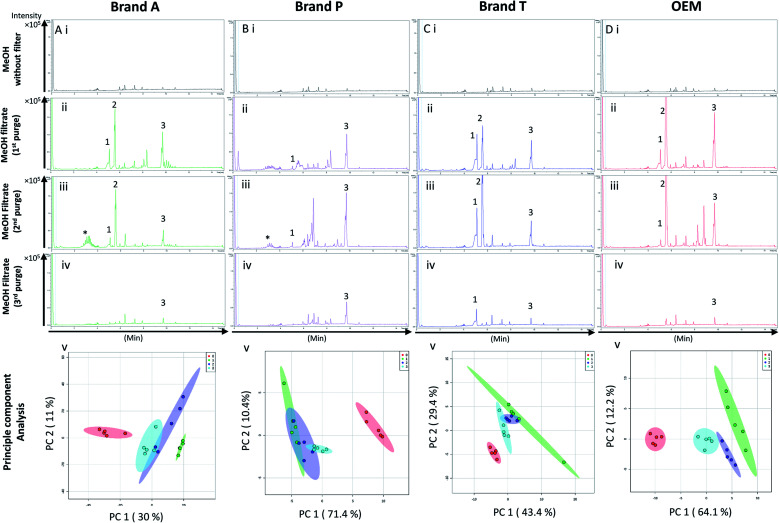
Base peak chromatograms and principle component analyses on PTFE membrane filter filtrates using various membrane filter brands at different purge intervals. Base peak chromatogram acquired from MeOH filtrates *via* (A) Brand A, (B) Brand P, (C) Brand T, and (D) OEM PTFE membrane filter. Profiled chromatogram of (i) unfiltered MeOH; (ii) MeOH filtrate at 1st purge; (iii) MeOH filtrates at 2nd purge; (iv) MeOH filtrates at 3rd purge. (v) Principle component analysis of filtrates at 3 different purge intervals against unfiltered MeOH. Red eclipse: unfiltered MeOH; green eclipse: 1st MeOH purge; blue eclipse: 2nd MeOH purge; turquoise eclipse: 3rd MeOH purge intervals.

**Fig. 3:**
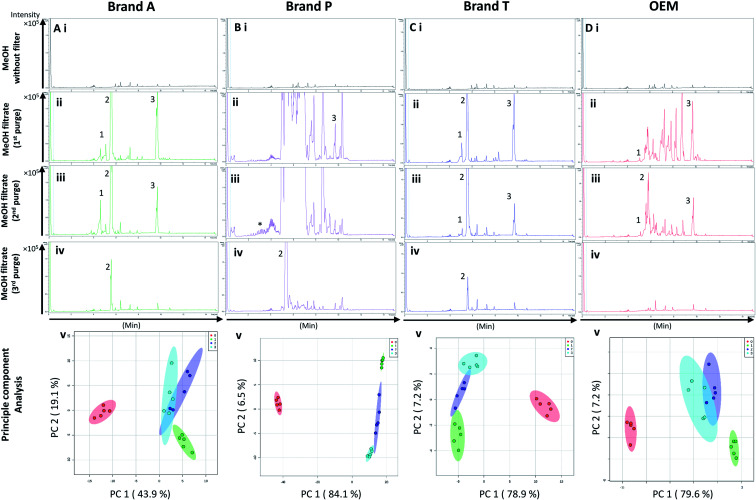
Base peak chromatograms and principle component analyses on PVDF membrane filter filtrates using various membrane filter brands at different purge intervals. Base peak chromatogram acquired from MeOH filtrates *via* (A) Brand A, (B) Brand P, (C) Brand T, and (D) OEM PTFE membrane filter. Profiled chromatogram of (i) unfiltered MeOH; (ii) MeOH filtrate at 1st purge; (iii) MeOH filtrates at 2nd purge; (iv) MeOH filtrates at 3rd purge. (v) Principle component analysis of filtrates at 3 different purge intervals against unfiltered MeOH. Red eclipse: unfiltered MeOH; green eclipse: 1st MeOH purge; blue eclipse: 2nd MeOH purge; turquoise eclipse: 3rd MeOH purge intervals.

High-throughput, unbiased profiling of MeOH (non-filtered) shows low (approximately 1.2 × 10^4^) but consistent presence of ghost peaks as shown in Fig. 2 and 3A–D(i). It is postulated that these ghost peaks were contributed by impurities eluted from the chromatographic system, including the analytical column, as it was used for chromatographic separation of more than 8000 samples prior to this study. Fig. 2 and 3A–D(ii) show the base-peak chromatograms (BPC) of MeOH filtered through different brands of fluoropolymer-based membrane filters (1^st^ purge). Extractable impurities were detected at varying degrees throughout the conditioning (purge) intervals. Spontaneous wavelength-based analysis revealed that the ghost peaks and extractable impurities were not ultraviolet-active as there were no peaks recorded when using different ultraviolet wavelengths ranging from 190–380 nm (Fig. S1†).

### 3.1 Detection of extractable impurities after filtration

Fluoropolymer-based membrane filters were reported to be compatible with a wide range of chemicals at different physical (pH and temperature) conditions.^24–26^ However, the mass spectrometry chromatograms showed that observable amounts of extractable impurities could be detected in the filtrates. For filtrates obtained using conditioned (1^st^ purge) membrane filters, polymer-like impurities were detected between 2–4 min in BPC of Brand A and P PTFE membranes filters (asterisk, Fig. 2A and B(ii)) whereas non-observable polymer-like signals were observed in remaining PTFE filters (Fig. 2C and D(ii)). Similarly, polymer-like signals were also observed in the Brand P PVDF membrane filter (Fig. 3B(ii)). However, the signal intensities of the extractable impurities present in Brand P PVDF membrane filter were noticeably higher compared to other brands' PVDF membrane filters as well as compared to all PTFE membrane filters (Fig. 3B(ii)). Following 2^nd^ purge, MeOH filtrates' BPC signals appeared to be enhanced (Fig. 2 and 3A–D(iii)). A post-sample blank injection was performed immediately after, and it was ascertained that these elevated signals from the impurities were not carried over from the earlier injections (data not shown). It is postulated that exposed total surface area (TSA) increased after first purging. An increase in TSA exposed to the sample/solvent causes more impurities to leech into the sample/solvent. As the number of purging increases, the adsorbed materials were flushed. This, in turn, leads to a cleaner chromatogram baseline as shown in both PTFE and PVDF filters at later purge intervals (Fig. 2 and 3A–D(iv)). Chemical structure of both PTFE and PVDF, both structures were proven to be chemically resistant,^27,28^ thus the breakdown or degradation of the PTFE and PVDF materials is not likely to happen. Thus, the extractable impurities may be due to the formulation used for the housing component, or a component introduced during the manufacturing process. It is suspected that the difference in severity of leaching between PTFE and PVDF is related to their respective surface chemistry. Based on Fig. 2 and 3A–D(iv), after the third purge, major eluted impurities were observed at retention times of 5 to 6 minutes. With reference to the mobile phase ratio, these impurities are likely to be semi-polar. The PVDF chain consists of alternating CH_2_ and CF_2_ groups, which possess strong dipoles. The fluorine atoms are unable to provide full protection for the carbon backbone, thus making the polymer polar overall.^29^ As such, it is relatively easier for polar or semi-polar external impurities to attack the electron density distribution of the PVDF membrane. This will result in a higher probability of forming polar interactions, such as dipole–dipole and dipole-induced dipoles interactions.^30,31^

There are peaks shared among different brands and different types of membrane filters materials (labelled as 1–3) as shown in Fig. 2 and 3. Tables 1 and 2 show a list of shared impurities across different brands of membrane filters. Identification of these leachable impurities is challenging using mass spectrometry (even using high-resolution mass spectrometry) because such technology could not distinguish structure chirality, isomer, isobaric and detailed molecules positioning. Although molecular fragmentation (MS/MS) could provide substantial information, the database holding extractable fluoropolymer-based membrane yet to be established. From the list of extractable impurities, we identified erucamide (*m*/*z* 337.3345)^32^ throughout the 8 different membrane filters where it matches the database with highest score. Erucamide is a slip agent that is commonly used to reduce the surface coefficient of friction, reduce static charge, lubrication of polymer during plastic fabrication.^33,34^ In order to confidently identify/elucidate vast numbers of impurities, employment of nuclear magnetic resonance on isolated impurities (up to micrograms per impurity) from the matrices is essential but perplexing. The reasons of impurities leeching from the membrane filter remained unknown. However, we deduced that these impurities eluted from the syringe filters are due to nonspecific binding of the formulation of housing component, or a component introduced during the manufacturing process.^34,35^ Apart from that, chemical additives added during polymer formulary for better plastic functionality and ageing properties of the polymer^34^ have a tendency to be eluted during the methanol purging.^34,35^ It was observed that the extractable impurities in the filtrates were reduced (Tables 1, 2, S1 and S2†) as the conditioning (number of purges) increased from 1 to 3. As such, choosing the right membrane filter with low nonspecific analyte binding, together with pre-flushing (pre-conditioning), is important so as to reduce the presence and amount of extractable impurities, especially for non-targeted studies. The presence of these extractable impurities can affect the interpretation of the analytical results.

**Table 1:** Shared extractable impurities from PTFE membrane between brands profiled using quadrupole-time-of flight coupled with ultra-high performance liquid chromatography^a^

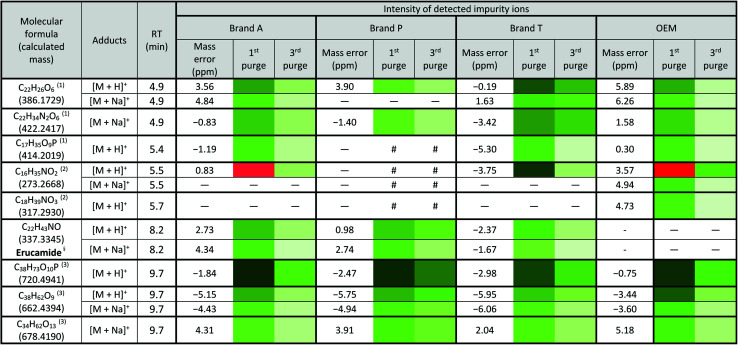

a Lowest intensity 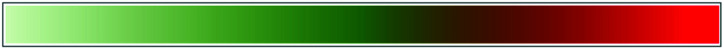 highest intensity. *Listed molecules are singly charged. ^#^The signal intensity is masked by the impurities which co-elute at the same retention time. — Not detected. (1), (2) & (3) are the peaks labelled in the chromatogram Fig. 2. ^§^Identified compound based on Keller *et al.*, 2008 (ref. 32) and its compound fragmentation was cross checked with MS-Finder.^21,22^

**Table 2:** Shared extractable impurities from PVDF membrane between brands profiled using quadrupole-time-of flight coupled with ultra-high performance liquid chromatography^a^

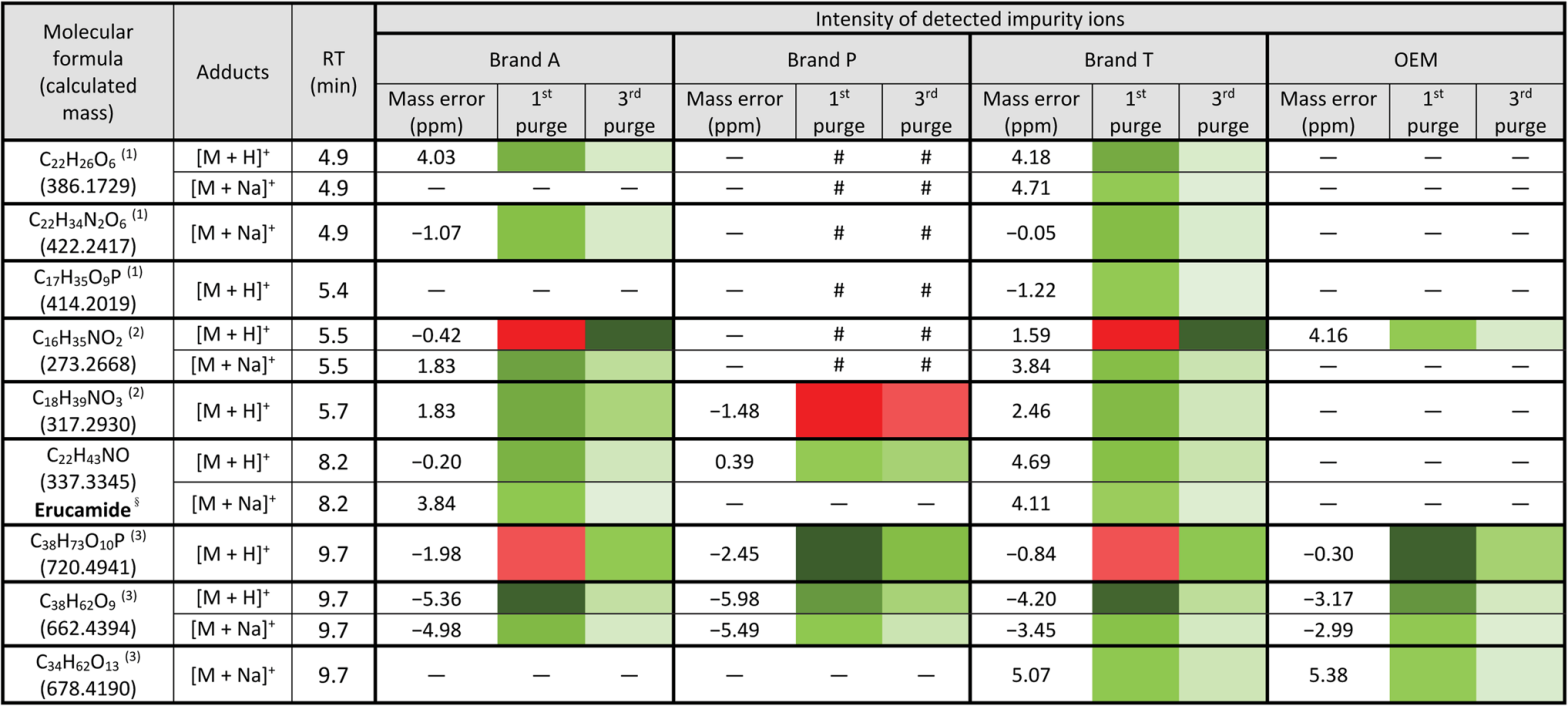

a Lowest intensity 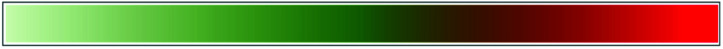 highest intensity. *Listed molecules are singly charged. ^#^The signal intensity is masked by the impurities which co-elute at the same retention time. — Not detected. (1), (2) & (3) are the peaks labelled in the chromatogram Fig. 3. ^§^Identified compound based on Keller *et al.*, 2008 (ref. 32) and its compound fragmentation was cross checked with MS-Finder.^21,22^

### 3.2 Unsupervised multivariate analysis on fluoropolymer-based membrane filtrates

Unsupervised multivariate analysis (principle component analysis) of the filtrates showed sizable eclipses (mean ± SD), indicating that there are variance between the replicates of the same filter brand (Fig. 2 and 3A–D(v)). Despite pre-conditioning the filters (purging up to 2 times) prior to use, the impurities still persist in the MeOH filtrates and these filtrates form clusters which are quite distant from the non-filtered MeOH cluster (Fig. 2 and 3A–D(v)). It was noted that filtrates obtained from filters pre-conditioned (purged) thrice (coloured in turquoise) exhibited lesser variance and the cluster tend to ‘migrate’ closer to the unfiltered MeOH cluster (coloured in red). The score plots revealed that the filtered groups contributed variances of between 30–71.4% and 43.9–84.1% along the principle component 1 on different brands of PTFE and PVDF membrane filters respectively (Fig. 2 and 3A–D(v)).

**Fig. 4:**
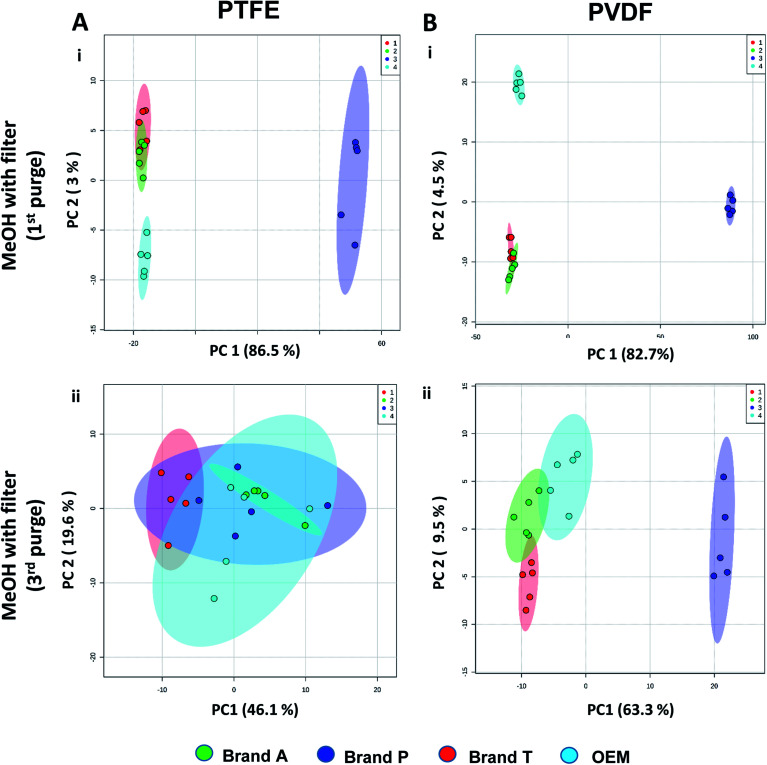
Principle component analyses between different brands membrane filter filtrates on (A) TFE and (B) PVDF at (i) 1st MeOH purge and (ii) 3rd MeOH purge intervals. Green eclipse: Brand A; blue eclipse: Brand P; red eclipse: Brand T; turquoise eclipse: OEM.

The PTFE membrane filtrates shared common contaminants (Tables 1 and 2) across different brands (Fig. 2A–D(i)–(iv)). However, additional impurities were present in Brand P filter as compared to other brands where peaks from those additional impurities were observed in Fig. 2B(ii)–(iv). These peaks contributed to the dissimilarity of the MeOH filtrates using Brand P filters as compare to the MeOH filtrates using other brand filters in principle component analysis (Fig. 4A(i), Table S1 and S2†). A similar observation can be made for filtrates collected from PVDF membrane filters (Fig. 4B(i), Table S2-2†), where Brand P's common peaks were masked by the presence of additional extractable impurities (Fig. 3A–D(ii) and (iii)). The results obtain seem to suggest that for high-throughput, untargeted analysis, sample filtration should be carried out using a single brand of filter since different extractable impurities from different brands of membrane filter could potentially contribute to false positive results.

The pre-conditioning (purging) of the PTFE membrane filters resulted in a reduction in the variances contributed by the differences in the PTFE membrane filter brands as the clusters tend to overlap between brands (Fig. 4Aii). In PVDF membrane filters, however, filtrates from Brand P formed a cluster which is relatively distant from filtrates obtained from other brands (Brand A, Brand T, and OEM) and that the other brands are clustered closer to each other (Fig. 4B(ii)). This outcome is expected due to the additional extractable impurities present in Brand P membrane filter (Fig. 3B(iv)). Although filtrates from PVDF membrane filter filtrates from Brand A, Brand T, and OEM generally clustered closer to the unfiltered MeOH (Fig. 3A(v), C(v), and D(v)) compared to filtrates from Brand P PVDF membrane filter (Fig. 3B(v)), those filtrates can still be distinguished from one another using PCA (Fig. 4B(ii)) due to the presence of extractable impurities in those brands. Membrane filter pre-conditioning was previously carried out by other researchers, though it was on nylon membrane filters. Up to 20 mL of MeOH was used to condition the nylon membrane filter, which resulted in a ∼10% reduction in the intensities contributed by the impurities.^11^ Based on the results obtained, the use of different membrane materials or different brands is not encouraged due to the presence of different types of impurities. The pre-conditioning of PTFE membrane filter is encouraged to reduce the false positives contributed by the extractable impurities. Extensive pre-conditioning, while could potentially further reduce the amount of extractable impurities, would not be practical and would defeat the purpose of these filters as they were designed to be ready-to-use out of the box. Excessive pre-conditioning would introduce another issue whereby it could potentially introduce microscopic tears, thus enabling submicron particles to pass though the said filter and interfere with subsequent analyses.

### 3.3 Evaluation of extractable interference on plant extract high-throughput analysis

**Fig. 5:**
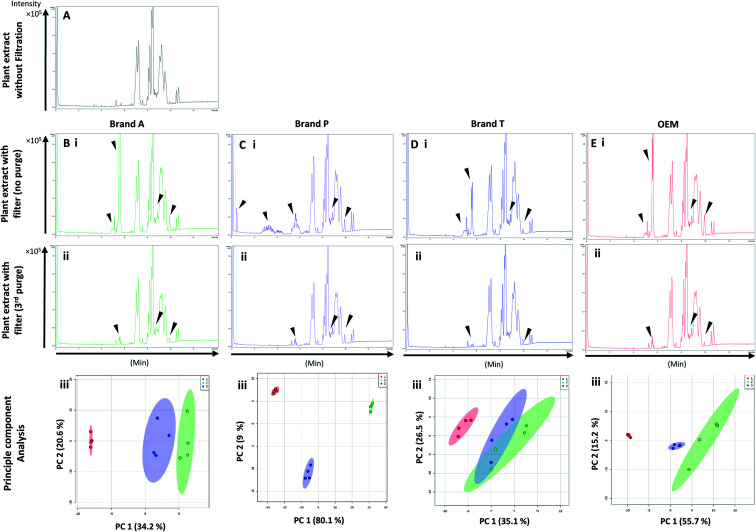
Base peak chromatograms and principle component analyses of *E. indica* nonpolar extract filtered through different brands of PTFE membrane filters. Base peak chromatogram of (A) unfiltered plant extract; (B) Brand A filtered plant extract; (C) Brand P filtered plant extract; (D) Brand T filtered plant extract; (E) OEM filtered plant extract. (i) Plant extract was filtered using an unconditioned (new) membrane filter; (ii) plant extract was filtered using 3-times conditioned membrane filter. (iii) Principle component analyses of filtrates against unfiltered *E. indica* non-polar extract. ▼ indicates consistent impurities peaks in Brand A, T and OEM filtrates. Red circle-unfiltered plant extract; green circle-extract filtered using unconditioned filter; blue circle-extract filtered using membrane filters that were pre-conditioned 3 times prior to use.

**Fig. 6:**
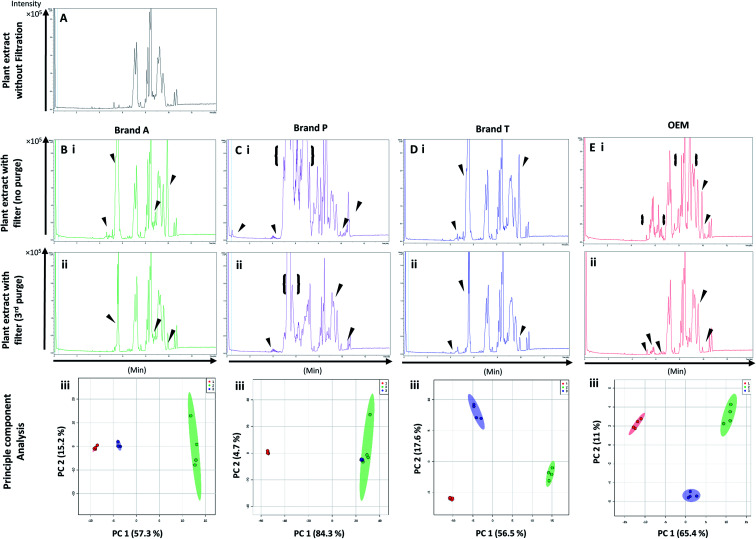
Base peak chromatograms and principle component analyses of *E. indica* non-polar extract filtered through different brands of PVDF membrane filters. Base peak chromatogram of (A) unfiltered plant extract; (B) Brand A filtered plant extract; (C) Brand P filtered plant extract; (D) Brand T filtered plant extract; (E) OEM filtered plant extract. (i) Plant extract was filtered using an unconditioned (new) filter membrane; (ii) plant extract was filtered using 3-times conditioned filter membrane. (iii) Principle component analyses of filtrates against unfiltered *E. indica* non-polar extract. ▼ indicates consistent impurities peaks in Brand A, T and OEM filtrates. {} indicates a wide range of signal-masking impurities extracted from Brand P membrane filter. Red circle-unfiltered plant extract; green circle-extract filtered using unconditioned filter; blue circle-extract filtered using membrane filters that were pre-conditioned 3 times prior to use.

Fig. 5 and 6 A show the base peak chromatograms of *E. indica* non-polar extract without the use of any membrane filters. Significant impurity peaks were observed throughout the chromatogram of the filtrate obtained from filtering the sample using fluoropolymer-based membrane filter without prior pre-conditioning. Filtrates from Brand P fluoropolymer membrane filters showed the most observable amount of impurities as compared to filtrates from other brands of fluoropolymer membrane filters (Fig. 5 and 6C(i) and (ii)). Peaks from impurities were observed in filtrates from Brand A, Brand T, and OEM membrane filters (marked with black inverted triangles). Pre-conditioning the membrane filter could reduce the amount of extractable impurities, though some of the impurities still remain in the filtrates as shown by the BPC. Filtering the sample using PVDF membrane filters without prior pre-conditioning (no purging) (Fig. 6B–E(iii)) showed obvious interference from the impurities as signals from the plant extract were masked by the signals from the said impurities. This was particularly evident in the filtrate whereby the plant extract was filtered using PVDF membrane filter from Brand P (Fig. 6C(ii)). Pre-conditioning the membrane filters prior to use could potentially reduce the interference coming from the said impurities, though the effects of the presence of the remaining impurities can affect multivariate analysis (Fig. 5 and 6B–E(iii)). This could potentially lead to false positive results.

## 4. Summary

Based on the results obtained, different brands syringe filters and membrane materials tend to elute different impurities at varying degrees. The signal intensities of the detected extractable impurities from the 2^nd^ purge interval (Fig. 2 and 3A–D(iii)) increased, possibly due to the membrane filter being disrupted by the MeOH that was filtered through the membrane filter earlier. The extractable impurities were noticeably reduced in PTFE membrane filters after the 3^rd^ purge, except for those from Brand P where noticeable amounts of extractable impurities are still present. The extractable impurities from PVDF membrane filters persist after 3-purges (Fig. 3A–D(iv)). Based on the calculated molecular formulae (Table S1 and S2†), the extractable impurities are not shared between different filter brands, as well as between different membrane filters (PVDF and PTFE). The base peak chromatograms (Fig. 2 and 3) showed that the intensities of the extractable impurities can be reduced by pre-conditioning the membrane filters, though PCA revealed that the filtrates obtained at different purge intervals are generally distant from the cluster formed by the unfiltered MeOH (Fig. 2 and 3A–D(v)). The co-clustering between different PTFE membrane filter brands, as shown in Fig. 4A, indicates that pre-conditioning of the membrane filters can reduce, but not eliminate, the dissimilarity between PTFE filter brands. Filtrates from different PVDF membrane filter brands tend to co-cluster according to their respective brands, thus suggesting that the dissimilarity between brands persist after pre-conditioning (Fig. 4B). As such, the pre-conditioning of PTFE membrane filters prior to use is encouraged to reduce the false positive signals contributed by the extractable impurities. The use of PVDF membrane filters in mass spectrometry based untargeted analysis is not encouraged as the extractable impurities from PVDF membrane filters persist even after the membrane filters were pre-conditioned 3 times. The use of different filter brands or different membrane filter materials (or a combination of both) during the filtration step is not encouraged due to the potentially different extractable impurities being eluted. A detailed and careful evaluation of the extractable impurities from membrane filters of different brands and materials should be conducted prior to switching brands or membrane filter material (or both) to avoid any complications during subsequent analyses.

## Conflicts of interest

There are no conflicts to declare.

## Supplementary Material

RA-009-C9RA06198C-s001

## References

[cit1] Trivedi D. K., Hollywood K. A., Goodacre R. (2017). New Horiz. Transl. Med..

[cit2] Roberts L. D. , Souza A. L., Gerszten R. E. and Clish C. B., Current protocols in molecular biology, 2012, vol. 98, pp. 1–24 10.1002/0471142727.mb3002s98 PMC3334318 22470063

[cit3] Xiao J. F., Zhou B., Ressom H. W. (2012). TrAC, Trends Anal. Chem..

[cit4] Feng Q., Liu Z., Zhong S., Li R., Xia H., Jie Z., Wen B., Chen X., Yan W., Fan Y., Guo Z., Meng N., Chen J., Yu X., Zhang Z., Kristiansen K., Wang J., Xu X., He K., Li G. (2016). Sci. Rep..

[cit5] Zampieri M., Sekar K., Zamboni N., Sauer U. (2017). Curr. Opin. Chem. Biol..

[cit6] Middleditch B. S., Zlatkis A. (1987). J. Chromatogr. Sci..

[cit7] Fritz J. S., Gjerde D. T., Becker R. M. (1980). Anal. Chem..

[cit8] Regnault C., Kano I., Darbouret D., Mabic S. (2004). J. Chromatogr. A.

[cit9] Lenk A. (2018). The Column.

[cit10] Hughes D. E., Bramer A. M. (1987). J. Chromatogr. A.

[cit11] Tran J. C., Doucette A. A. (2006). J. Am. Soc. Mass Spectrom..

[cit12] Williams S. (2004). J. Chromatogr. A.

[cit13] Stoll D. R. (2018). LCGC North Am..

[cit14] De Vos R. C. H., Moco S., Lommen A., Keurentjes J. J. B., Bino R. J., Hall R. D. (2007). Nat. Protoc..

[cit15] Guo N., Yang D., Liu C., Yan H., Yang X., Wang X., Fan B. (2019). Nat. Prod. Res..

[cit16] Bertrand S., Schumpp O., Bohni N., Bujard A., Azzollini A., Monod M., Gindro K., Wolfender J.-L. (2013). J. Chromatogr. A.

[cit17] Moco S., Bino R. J., Vorst O., Verhoeven H. A., de Groot J., van Beek T. A., Vervoort J., de Vos C. H. R. (2006). Plant Physiol..

[cit18] Pluskal T., Castillo S., Villar-Briones A., Orešič M. (2010). BMC Bioinf..

[cit19] Chong J., Soufan O., Li C., Caraus I., Li S., Bourque G., Wishart D. S., Xia J. (2018). Nucleic Acids Res..

[cit20] Xia J., Wishart D. S. (2016). Curr. Protoc. Bioinf..

[cit21] Tsugawa H., Kind T., Nakabayashi R., Yukihira D., Tanaka W., Cajka T., Saito K., Fiehn O., Arita M. (2016). Anal. Chem..

[cit22] Lai Z., Tsugawa H., Wohlgemuth G., Mehta S., Mueller M., Zheng Y., Ogiwara A., Meissen J., Showalter M., Takeuchi K., Kind T., Beal P., Arita M., Fiehn O. (2017). Nat. Methods.

[cit23] Ling Y. S., Lim L. R., Yong Y. S., Tamin O., Puah P. Y. (2018). Nat. Prod. Res..

[cit24] Gardiner J. (2015). Aust. J. Chem..

[cit25] Okazoe T. (2009). Proc. Jpn. Acad., Ser. B.

[cit26] Cardoso V. F., Correia D. M., Ribeiro C., Fernandes M. M., Lanceros-Méndez S. (2018). Polymers.

[cit27] Feng S., Zhong Z., Wang Y., Xing W., Drioli E. (2018). J. Membr. Sci..

[cit28] Liu F., Hashim N. A., Liu Y., Abed M. R. M., Li K. (2011). J. Membr. Sci..

[cit29] Johnson R. A. and Nguyen M. H., Properties of Macroporous Hydrophobic Membranes, Wiley & Sons, 2017, pp. 107–138

[cit30] Rehman W. U., Muhammad A., Younas M., Wu C., Hu Y., Li J. (2019). J. Membr. Sci..

[cit31] Lee S., Park J.-S., Lee T. R. (2008). Langmuir.

[cit32] Keller B. O., Sui J., Young A. B., Whittal R. M. (2008). Anal. Chim. Acta.

[cit33] Bhunia K., Sablani S. S., Tang J., Rasco B. (2013). Compr. Rev. Food Sci. Food Saf..

[cit34] Hahladakis J. N., Velis C. A., Weber R., Iacovidou E., Purnell P. (2018). J. Hazard. Mater..

[cit35] Liu L., Randolph T. W., Carpenter J. F. (2012). J. Pharm. Sci..

