# Supplementary material for: Extractable impurities from fluoropolymer-based membrane filters – interference in high-throughput, untargeted analysis
Source: RSC Adv. 2019 Oct 8;9(55):31918–27. doi: 10.1039/c9ra06198c (PMC9116114; doi:10.1039/c9ra06198c)
Supplement: RA-009-C9RA06198C-s001 [file RA-009-C9RA06198C-s001.pdf]

Electronic Supplementary Material (ESI) for RSC Advances.
This journal is © The Royal Society of Chemistry 2019

Electronic Supplementary Information (ESI)

Extractable Impurities from Fluoropolymer-Based Membrane Filters – Interference in High-throughput, Untargeted Analysis

Perng Yang **Puah**[‡a], Dexter Jiunn Herng **Lee**[‡b], Ken Hing **Mak**[c], Hui Jun **Ang**[c], Hsing-Chang **Chen**[d], Pak Yan **Moh**[e,f], Siat Yee **Fong***[a] and Yee Soon **Ling***[f]

[a]Faculty of Medicine and Health Sciences, Universiti Malaysia Sabah, Jalan UMS, 88400 Kota Kinabalu, Sabah, Malaysia

[b]Biotechnology Research Institute, Universiti Malaysia Sabah, Jalan UMS, 88400 Kota Kinabalu, Sabah, Malaysia

[c]Faculty of Sustainable Agriculture, Universiti Malaysia Sabah Locked Bag No 3, 90509, Sandakan, Sabah, Malaysia

[d]Institute of Food Safety and Health, National Taiwan University, No. 17, Xu-Zhou Rd., Taipei, Taiwan 10055

[e]Faculty of Sciences and Natural Resources, Universiti Malaysia Sabah, Jalan UMS, 88400 Kota Kinabalu, Sabah, Malaysia

[f]Water Research Unit, Universiti Malaysia Sabah, Jalan UMS, 88400 Kota Kinabalu, Sabah, Malaysia

‡These authors contributed equally to this work.
*Corresponding author: siatyee@ums.edu.my; ling82ys@gmail.com

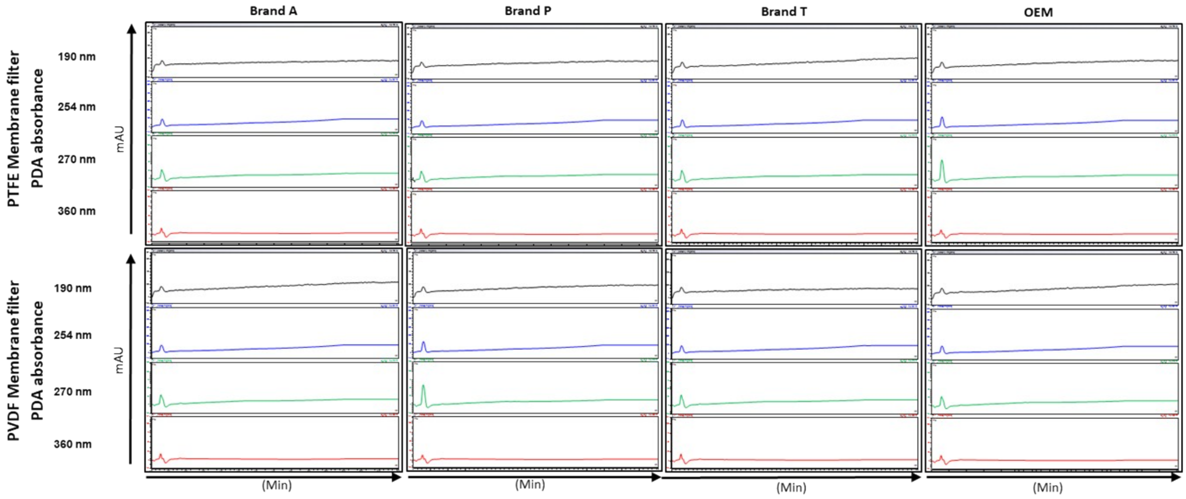

Figure S1: 1st purge of MeOH filtrates eluted from different brands scanned using different ultraviolet wavelengths ranging from 190-380 nm.

Table S 1-1: Detected extractable impurities from **Brand A, PTFE** membrane filter using Quadrupole time of flight coupled with ultra-high performance liquid chromatography.

Detected *m/z**	RT (min)	Tentative adducts	Molecular formulae	Mass error (ppm)	Signals intensities			Kruskal-Wallis (*p*-value)[a]	Fold-changes [b]
					1st purge	3rd purge	Reduction of impurities, %		
387.1788	4.9	$[M+H]^+$	$C_{22} H_{26} O_6$	3.56	24992	2837	88.7	●	-1.35
409.1603	4.9	$[M+Na]^+$	$C_{22} H_{26} O_6$	4.84	7704	1139	85.2	●●●	-1.28
445.2313	4.9	$[M+Na]^+$	$C_{22} H_{34} N_2 O_6$	-0.83	17659	2590	85.3	●	-1.14
415.2096	5.3	$[M+H]^+$	$C_{17} H_{35} O_9 P$	-1.19	9760	1046	89.3	●●●	-1.55
473.2624	5.3	$[M+H]^+$	$C_{26} H_{36}$ N2 O_6	4.62	11381	1602	85.9	●●●	-1.71
274.2738	5.5	$[M+H]^+$	$C_{16} H_{35} N O_2$	0.83	107560	2757	97.4	●●●	-2.41
338.3408	8.2	$[M+H]^+$	$C_{22} H_{43} N O$	2.73	18883	2042	89.2	●	-1.48
360.3222	8.2	$[M+Na]^+$	$C_{22} H_{43} N O$	4.34	8993	355	96.1	●●●	-2.40
721.5027	9.7	$[M+H]^+$	$C_{38} H_{73} O_{10} P$	-1.84	57613	7185	87.5	●●●	-1.74
663.4501	9.7	$[M+H]^+$	$C_{38} H_{62} O_9$	-5.15	22969	2442	89.4	●●●	-1.25
701.4053	9.7	$[M+Na]^+$	$C_{34} H_{62} O_{13}$	4.31	6471	1459	77.5	●●	-0.94
685.4315	9.7	$[M+Na]^+$	$C_{38} H_{62} O_9$	-4.43	7793	1054	86.5	●●	-1.65
718.2898	10.0	$[M+NH_4]^+$	$C_{32} H_{44} O_{17}$	2.66	10226	1327	87.0	●●●	-1.63
702.3159	10.1	$[M+NH_4]^+$	$C_{36} H_{44} O_{13}$	-5.62	12213	1046	91.4	●	-2.02
697.3613	10.1	$[M+H]^+$	$C_{39} H_{52} O_{11}$	-4.44	14749	796	94.6	●●	-2.21
746.3207	10.2	$[M+NH_4]^+$	$C_{41} H_{44} O_{12}$	-4.88	10017	2380	76.2	●●	-0.83
725.3921	10.3	$[M+Na]^+$	$C_{39} H_{58} O_{11}$	-7.03	13775	1148	91.7	●	-2.01
730.3476	10.3	$[M+Na]^+$	$C_{36} H_{53} N O_{13}$	-9.50	11600	1765	84.8	●●●	-1.61
753.4235	10.4	$[M+H]^+$	$C_{36} H_{64} O_{16}$	4.33	7846	926	88.2	●●	-1.69
774.3522	10.4	$[M+H]^+$	$C_{31} H_{55}$ N3 O_{19}	-2.48	8024	1997	75.1	●●	-0.81

* Listed molecules are single-charged

[a]●, *p*-value < 0.05; ●●, *p*-value < 0.01; ●●●, *p*-value < 0.001 determined by parametric Kruskal-Wallis test

[b]Fold changes of peak area relative to mean control. Log_2 transformed. Values > 0 represent increase relative to 1st purge and values < 0 represent decrease relative to 1st purge

Table S 1-2: Detected extractable impurities from **Brand P, PTFE** membrane filter using Quadrupole-time-of flight coupled with ultra-high performance liquid chromatography.

**Detected *m/z****	**RT (min)**	**Tentative adducts**	**Molecular formulae**	**Mass error (ppm)**	**Signals intensities**			**Kruskal-Wallis (*p*-value)[a]**	**Fold-changes [b]**
					**1st purge**	**3rd purge**	**Reduction of impurities, %**		
172.09465	0.5	$[M+Na]^+$	$C_6 H_{15} N O_3$	-1.58	6244	267	95.7	●●●	-4.99
150.11279	0.5	$[M+H]^+$	$C_6 H_{15} N O_3$	-2.15	33442	1086	96.8	●●●	-4.95
132.10245	0.6	$[M+H]^+$	$C_6 H_{13} N O_2$	-4.16	7018	204	97.1	●●●	-3.21
166.10751	0.6	$[M+NH_4]^+$	$C_6 H_{12} O_4$	-0.85	5501	131	97.6	●●●	-6.20
387.17871	4.9	$[M+H]^+$	$C_{22} H_{26} O_6$	3.90	5836	2193	62.4	●	-1.21
445.23150	4.9	$[M+Na]^+$	$C_{22} H_{34} N_2 O_6$	-1.40	5697	2044	64.1	●●	-1.39
547.32206	5.3	$[M+NH_4]^+$	$C_{27} H_{39} N_5 O_6$	3.40	7913	158	98.0	●●●	-5.59
591.34787	5.4	$[M+H]^+$	$C_{33} H_{50} O_9$	8.28	7715	157	98.0	●●●	-5.80
635.37277	5.5	$[M+Na]^+$	$C_{33} H_{56} O_{10}$	6.20	5585	116	97.9	●●●	-6.88
338.34141	8.2	$[M+H]^+$	$C_{22} H_{43} N O$	0.98	17356	5924	65.9	●	-1.42
360.32276	8.2	$[M+Na]^+$	$C_{22} H_{43} N O$	2.74	7938	2363	70.2	●●●	-1.56
721.50319	9.7	$[M+H]^+$	$C_{38} H_{73} O_{10} P$	-2.47	55072	34085	38.1	●●	-1.34
663.45047	9.7	$[M+H]^+$	$C_{38} H_{62} O_9$	-5.75	22178	12156	45.2	●	-1.44
701.40561	9.7	$[M+Na]^+$	$C_{34} H_{62} O_{13}$	3.91	7140	4832	32.3	●●●	-1.40
685.43188	9.7	$[M+Na]^+$	$C_{38} H_{62} O_9$	-4.94	7627	4424	42.0	●●●	-2.02

* Listed molecules are single-charged

[a]●, *p*-value < 0.05; ●●, *p*-value < 0.01; ●●●, *p*-value < 0.001 determined by parametric Kruskal-Wallis test

[b]Fold changes of peak area relative to mean control. Log_2 transformed. Values > 0 represent increase relative to 1st purge and values < 0 represent decrease relative to 1st purge

Table S 1-3: Detected extractable impurities from **Brand T, PTFE** membrane filter using Quadrupole time of flight coupled with ultra-high performance liquid chromatography.

**Detected *m/z****	**RT (min)**	**Tentative adducts**	**Molecular formulae**	**Mass error (ppm)**	**Signals intensities**			**Kruskal-Wallis (*p*-value)[a]**	**Fold-changes [b]**
					**1st purge**	**3rd purge**	**Reduction of impurities, %**		
445.2324	4.9	$[M+Na]^+$	$C_{22}H_{34}N_2O_6$	-3.42	27139	16528	39.1	●●	-1.56
387.1803	4.9	$[M+H]^+$	$C_{22}H_{26}O_6$	-0.19	42488	20175	52.5	●●●	-1.75
409.1615	4.9	$[M+Na]^+$	$C_{22}H_{26}O_6$	1.63	12694	7525	40.7	●●●	-1.82
425.1353	5.0	$[M+H]^+$	$C_{22}H_{20}N_2O_7$	-2.36	6729	5267	21.7	●	-0.38
415.2113	5.4	$[M+H]^+$	$C_{17}H_{35}O_9P$	-5.30	9428	409	95.7	●●●	-1.79
473.2638	5.5	$[M+H]^+$	$C_{26}H_{36}N_2O_6$	1.70	11429	594	94.8	●●●	-1.69
274.2751	5.5	$[M+H]^+$	$C_{16}H_{35}NO_2$	-3.75	71064	2439	96.6	●●●	-3.19
338.3425	8.2	$[M+H]^+$	$C_{22}H_{43}NO$	-2.37	10116	1907	81.2	●	-1.01
360.3243	8.2	$[M+Na]^+$	$C_{22}H_{43}NO$	-1.67	5105	314	93.9	●●	-0.64
721.5036	9.7	$[M+H]^+$	$C_{38}H_{73}O_{10}P$	-2.98	46259	13268	71.3	●	-1.17
663.4506	9.7	$[M+H]^+$	$C_{38}H_{62}O_9$	-5.95	18813	4420	76.5	●●	-1.25
701.4069	9.7	$[M+Na]^+$	$C_{34}H_{62}O_{13}$	2.04	6417	2520	60.7	●	-0.67
685.4326	9.7	$[M+Na]^+$	$C_{38}H_{62}O_9$	-6.06	6515	1915	70.6	●●	-0.87

* Listed molecules are single-charged

[a]●, *p*-value < 0.05; ●●, *p*-value < 0.01; ●●●, *p*-value < 0.001 determined by parametric Kruskal-Wallis test

[b]Fold changes of peak area relative to mean control. Log_2 transformed. Values > 0 represent increase relative to 1st purge and values < 0 represent decrease relative to 1st purge

Table S 1-4: Detected extractable impurities from **OEM,** **PTFE** membrane filter using Quadrupole time of flight coupled with ultra-high performance liquid chromatography.

Detected *m/z**	RT (min)	Tentative adducts	Molecular formulae	Mass error (ppm)	Signals intensities			Kruskal-Wallis (*p*-value)[a]	Fold-changes [b]
					1st purge	3rd purge	Reduction of impurities, %		
387.1779	4.9	$[M+H]^+$	$C_{22} H_{26} O_6$	5.89	23384	745	96.8	●●●	-1.07
445.2302	4.9	$[M+Na]^+$	$C_{22} H_{34} N_2 O_6$	1.58	15939	726	95.5	●●	-0.80
409.1597	4.9	$[M+Na]^+$	$C_{22} H_{26} O_6$	6.26	7330	331	95.5	●●	-1.45
437.1907	5.4	$[M+NH_4]^+$	$C_{21} H_{25} N O_8$	2.84	7154	139	98.1	●●●	-2.83
415.2090	5.4	$[M+H]^+$	$C_{17} H_{35} O_9 P$	0.30	10119	223	97.8	●●●	-1.56
473.2615	5.5	$[M+H]^+$	$C_{26} H_{36} N_2 O_6$	6.51	12350	345	97.2	●●	-1.41
274.2731	5.5	$[M+H]^+$	$C_{16} H_{35} N O_2$	3.57	618291	6833	98.9	●●●	-2.76
296.2547	5.5	$[M+Na]^+$	$C_{16} H_{35} N O_2$	4.94	11874	312	97.4	●	-1.73
230.2471	5.7	$[M+H]^+$	$C_{14} H_{31} N O$	3.10	7311	153	97.9	●●●	-2.41
318.2988	5.7	$[M+H]^+$	$C_{18} H_{39} N O_3$	4.73	7432	160	97.9	●●	-2.01
492.4018	8.2	$[M+NH_4]^+$	$C_{30} H_{50} O_4$	6.21	7397	NA	100.0	-	-
520.4332	8.8	$[M+NH_4]^+$	$C_{32} H_{54} O_4$	5.72	14659	179	98.8	●●●	-1.36
721.5020	9.7	$[M+H]^+$	$C_{38} H_{73} O_{10} P$	-0.75	88231	10157	88.5	●●	-1.11
663.4489	9.7	$[M+H]^+$	$C_{38} H_{62} O_9$	-3.44	40390	3520	91.3	●●●	-0.98
701.4048	9.7	$[M+Na]^+$	$C_{34} H_{62} O_{13}$	5.18	8915	1944	78.2	●●	-0.87
685.4310	9.7	$[M+Na]^+$	$C_{38} H_{62} O_9$	-3.60	11047	1495	86.5	●●●	-1.02

* Listed molecules are single-charged

[a]●, *p*-value < 0.05; ●●, *p*-value < 0.01; ●●●, *p*-value < 0.001 determined by parametric Kruskal-Wallis test

[b]Fold changes of peak area relative to mean control. Log_2 transformed. Values > 0 represent increase relative to 1st purge and values < 0 represent decrease relative to 1st purge

Table S 2-1: Detected extractable impurities from **Brand A, PVDF** membrane filter using Quadrupole-time-of flight coupled with ultra-high performance

Detected *m/z**	RT (min)	Tentative adducts	Molecular formulae	Mass error (ppm)	Signals intensities			Kruskal-Wallis (*p*-value)[a]	Fold-changes [b]
					1st purge	3rd purge	Reduction of impurities, %		
568.29327	4.5	$[M+H]^+$	$C_{21}H_{41}N_7O_{11}$	0.73	16384	2957	81.95	●●●	-1.21
445.23136	4.9	$[M+Na]^+$	$C_{22}H_{34}N_2O_6$	-1.07	13803	1066	92.28	●●	-1.21
387.17866	4.9	$[M+H]^+$	$C_{22}H_{26}O_6$	4.03	19728	1131	94.27	●●●	-1.86
296.25550	5.5	$[M+Na]^+$	$C_{16}H_{35}NO_2$	1.83	26579	3221	87.88	●●●	-1.31
274.27417	5.5	$[M+H]^+$	$C_{16}H_{35}NO_2$	-0.42	1428802	85918	93.99	●●	-1.81
230.24798	5.6	$[M+H]^+$	$C_{14}H_{31}NO$	-0.61	99261	2580	97.40	●●●	-1.42
318.29969	5.7	$[M+H]^+$	$C_{18}H_{39}NO_3$	1.83	22997	4727	79.45	●●●	-1.11
338.34181	8.2	$[M+H]^+$	$C_{22}H_{43}NO$	-0.20	19671	1671	91.51	●●●	-1.72
360.32239	8.2	$[M+Na]^+$	$C_{22}H_{43}NO$	3.84	9364	285	96.96	●●●	-1.74
665.44103	9.5	$[M+H]^+$	$C_{34}H_{65}O_{10}P$	-3.34	7563	343	95.46	●●	-1.53
721.50284	9.7	$[M+H]^+$	$C_{38}H_{73}O_{10}P$	-1.98	152351	8306	94.55	●●	-1.44
663.45021	9.7	$[M+H]^+$	$C_{38}H_{62}O_9$	-5.36	75075	2802	96.27	●●	-1.71
685.43190	9.7	$[M+Na]^+$	$C_{38}H_{62}O_9$	-4.98	17092	1231	92.80	●●	-1.06

liquid chromatography.

* Listed molecules are single-charged

[a]●, *p*-value < 0.05; ●●, *p*-value < 0.01; ●●●, *p*-value < 0.001 determined by parametric Kruskal-Wallis test

[b]Fold changes of peak area relative to mean control. Log_2 transformed. Values > 0 represent increase relative to 1st purge and values < 0 represent decrease relative to 1st purge

Table S 2-2: Detected extractable impurities from **Brand P, PVDF** membrane filter using Quadrupole-time-of flight coupled with ultra-high performance liquid chromatography.

* Listed molecules are single-charged

[a]●, *p*-value < 0.05; ●●, *p*-value < 0.01; ●●●, *p*-value < 0.001 determined by parametric Kruskal-Wallis test

**Detected *m/z****	**RT (min)**	**Tentative adducts**	**Molecular formulae**	**Mass error (ppm)**	**Signals intensities**			**Kruskal-Wallis (*p*-value)[a]**	**Fold-changes [b]**
					**1st purge**	**3rd purge**	**Reduction of impurities, %**		
164.12852	0.5	$[M+H]^+$	$C_7 H_{17} N O_3$	-2.45	8417	306	96.36	●●●	-2.21
178.14397	0.5	$[M+NH_4]^+$	$C_8 H^{16} O_3$	-1.25	10712	391	96.35	●●●	-1.31
162.14914	0.7	$[M+H]^+$	$C_8 H_{19} N O_2$	-1.77	5089	195	96.17	●	-2.14
192.15975	0.8	$[M+H]^+$	$C_9 H_{21} N O_3$	-1.73	10088	1298	87.13	●	-1.63
304.28455	4.9	$[M+NH_4]^+$	$C_{17} H_{34} O_3$	0.25	183484	10164	94.46	●●●	-1.26
316.28423	5.0	$[M+H]^+$	$C_{18} H_{37} N O_3$	1.24	14408	847	94.12	●●●	-1.21
387.17928	5.1	$[M+NH_4]^+$	$C_{23} H_{19} N_3 O_2$	6.16	6590	1757	73.34	●	-0.84
635.59063	5.3	$[M+H]^+$	$C_{32} H_{74} N_8 O_4$	-0.08	47044	N/A	100.00	-	-
362.32605	5.4	$[M+H]^+$	$C_{20} H_{43} N O_4$	1.20	87885	11691	86.70	●●●	-0.69
288.28976	5.5	$[M+H]^+$	$C_{17} H_{37} N O_2$	-0.19	11067	1450	86.90	●	-0.93
332.31577	5.5	$[M+NH_4]^+$	$C_{19} H_{38} O_3$	0.48	546457	35512	93.50	●●●	-1.58
318.30074	5.7	$[M+H]^+$	$C_{18} H_{39} N O_3$	-1.48	2724822	434688	84.05	●●●	-1.20
321.26317	5.7	$[M+Na]^+$	$C_{16} H_{34} N_4 O$	-2.31	48521	1052	97.83	●●●	-1.57
470.36726	5.7	$[M+Na]^+$	$C_{22} H_{49} N_5 O_4$	0.93	72748	3799	94.78	●●	-1.95
514.39298	5.8	$[M+Na]^+$	$C_{24} H_{53} N_5 O_5$	1.85	53396	3362	93.70	●●●	-1.62
484.38262	6.0	$[M+Na]^+$	$C_{23} H_{51} N_5 O_4$	1.53	157977	6307	96.01	●●●	-1.79
528.40850	6.0	$[M+Na]^+$	$C_{25} H_{55} N_5 O_5$	2.06	131285	7377	94.38	●●	-1.88
335.27886	6.1	$[M+Na]^+$	$C_{17} H_{36} N_4 O$	-2.33	63868	972	98.48	●●●	-1.82
572.43424	6.1	$[M+Na]^+$	$C_{27} H_{59} N_5 O_6$	2.76	103108	7355	92.87	●	-1.59
616.45990	6.2	$[M+NH_4]^+$	$C_{37} H_{58} O_6$	-4.57	76127	6710	91.19	●	-1.48
349.29432	6.4	$[M+H]^+$	$C_{19} H_{40} O_5$	1.52	66578	1369	97.94	●●●	-1.81
377.32540	6.5	$[M+Na]^+$	$C_{20} H_{42} N_4 O$	-0.90	271253	6976	97.43	●●●	-1.89
586.44980	6.6	$[M+NH_4]^+$	$C_{36} H_{56} O_5$	-5.63	80630	5337	93.38	●●	-1.76
630.47551	6.6	$[M+NH_4]^+$	$C_{38} H_{60} O_6$	-4.40	57057	5105	91.05	●●	-1.11
333.29962	6.9	$[M+H]^+$	$C_{19} H_{40} O_4$	0.95	65947	1539	97.67	●●	-1.53

614.48077	6.8	$[M+NH_4]^+$	$C_{38}H_{60}O_5$	-4.81	142766	12653	91.14	●●●	-1.93
658.50671	6.8	$[M+NH_4]^+$	$C_{41}H_{60}N_4O_2$	-1.96	98583	10203	89.65	●●●	-0.71
702.53250	7.0	$[M+NH_4]^+$	$C_{42}H_{68}O_7$	-3.17	64635	7678	88.12	●●●	-1.02
353.26556	7.1	$[M+Na]^+$	$C_{19}H_{38}O_4$	2.03	34589	3979	88.50	●	-0.63
359.31493	7.8	$[M+H]^+$	$C_{21}H_{42}O_4$	1.83	11045	485	95.61	●●●	-2.35
341.30453	7.9	$[M+H]^+$	$C_{21}H_{40}O_3$	1.44	17312	800	95.38	●●	-2.26
381.29675	7.5	$[M+Na]^+$	$C_{21}H_{42}O_4$	2.18	85952	10250	88.07	●	-0.97
267.26817	7.6	$[M+H]^+$	$C_{18}H_{34}O$	0.27	8024	431	94.63	●●	-1.93
695.35529	7.8	$[M+H]^+$	$C_{38}H_{50}N_2O_{10}$	-2.11	10634	1717	83.85	●	-2.79
338.34161	8.2	$[M+H]^+$	$C_{22}H_{43}NO$	0.39	6973	5128	26.46	●	-0.32
738.38508	8.3	$[M+Na]^+$	$C_{28}H_{57}N_7O_{14}$	0.69	6924	2554	63.11	●	-0.72
458.45521	8.4	$[M+NH_4]^+$	$C_{28}H_{56}O_3$	3.54	7113	368	94.83	●●	-2.03
472.47111	8.6	$[M+NH_4]^+$	$C_{29}H_{58}O_3$	2.89	225560	9729	95.69	●●●	-2.68
679.36023	8.6	$[M+Na]^+$	$C_{37}H_{48}N_6O_5$	-3.64	5801	1443	75.12	●●	-1.42
486.48655	8.8	$[M+NH_4]^+$	$C_{30}H_{60}O_3$	3.25	36366	427	98.83	●●●	-1.82
663.45062	9.7	$[M+H]^+$	$C_{38}H_{62}O_9$	-5.98	30511	4946	83.79	●●●	-1.68
721.50318	9.7	$[M+H]^+$	$C_{38}H_{73}O_{10}P$	-2.45	68094	14886	78.14	●●	-1.64
685.43224	9.7	$[M+Na]^+$	$C_{38}H_{62}O_9$	-5.49	9095	2113	76.77	●●	-1.62
579.53232	10.1	$[M+H]^+$	$C_{37}H_{70}O_4$	4.09	27367	3934	85.63	●●●	-1.98
619.52456	10.1	$[M+Na]^+$	$C_{37}H_{72}O_5$	4.42	30693	13111	57.28	●	-0.47
607.56350	10.3	$[M+H]^+$	$C_{39}H_{74}O_4$	4.10	219735	24254	88.96	●●	-0.85
647.55565	10.3	$[M+Na]^+$	$C_{39}H_{76}O_5$	4.56	54029	25821	52.21	●	-1.13
642.60004	10.4	$[M+NH_4]^+$	$C_{39}H_{76}O_5$	4.90	16579	547	96.70	●●●	-1.84

[b]Fold changes of peak area relative to mean control. Log_2 transformed. Values > 0 represent increase relative to 1st purge and values < 0 represent decrease relative to 1st purge

Table S2-3: Detected extractable impurities from **Brand T, PVDF** membrane filter using Quadrupole-time-of flight coupled with ultra-high performance liquid chromatography.

**Detected *m/z****	**RT (min)**	**Tentative adducts**	**Molecular formulae**	**Mass error (ppm)**	**Signals intensities**			**Kruskal-Wallis (*p*-value)[a]**	**Fold-changes [b]**
					**1st purge**	**3rd purge**	**Reduction of impurities, %**		
409.16034	4.9	$[M+Na]^+$	$C_{22}H_{26}O_6$	4.71	7487	380	94.92	●●	-1.69
445.23093	4.9	$[M+Na]^+$	$C_{22}H_{34}$ N2 O_6	-0.05	15085	854	94.34	●●	-1.72
387.17860	4.9	$[M+H]^+$	$C_{22}H_{26}O_6$	4.18	22440	939	95.82	●●●	-1.80
415.20965	5.4	$[M+H]^+$	$C_{17}H_{35}O_9P$	-1.22	7862	198	97.48	●●●	-1.81
473.26251	5.3	$[M+Na]^+$	$C_{22}H_{43}O_7P$	3.00	9429	257	97.27	●	-1.87
296.25495	5.5	$[M+Na]^+$	$C_{16}H_{35}NO_2$	3.84	14430	2187	84.84	●●●	-1.52
437.19151	5.5	$[M+NH_4]^+$	$C_{21}H_{25}NO_8$	0.79	6263	147	97.65	●●●	-1.78
274.27362	5.5	$[M+H]^+$	$C_{16}H_{35}NO_2$	1.59	806372	53297	93.39	●●	-1.55
230.24763	5.6	$[M+H]^+$	$C_{14}H_{31}NO$	0.92	17558	763	95.65	●●●	-1.64
318.29949	5.7	$[M+H]^+$	$C_{18}H_{39}NO_3$	2.46	16126	1721	89.33	●●●	-0.89
338.34016	8.2	$[M+H]^+$	$C_{22}H_{43}NO$	4.69	13743	2284	83.38	●●●	-1.66
360.32230	8.2	$[M+Na]^+$	$C_{22}H_{43}NO$	4.11	6262	651	89.60	●	-0.92
663.44944	9.7	$[M+H]^+$	$C_{38}H_{62}O_9$	-4.20	51440	3240	93.70	●●●	-1.14
701.40482	9.7	$[M+Na]^+$	$C_{34}H_{62}O_{13}$	5.07	9317	1923	79.36	●●	-0.69
685.43089	9.7	$[M+Na]^+$	$C_{38}H_{62}$ O9	-3.45	12762	1351	89.41	●	-1.61
721.50202	9.7	$[M+H]^+$	$C_{38}H_{73}O_{10}P$	-0.84	106323	9649	90.92	●●●	-0.99

* Listed molecules are single-charged

[a]●, *p*-value < 0.05; ●●, *p*-value < 0.01; ●●●, *p*-value < 0.001 determined by parametric Kruskal-Wallis test

[b]Fold changes of peak area relative to mean control. Log_2 transformed. Values > 0 represent increase relative to 1st purge and values < 0 represent decrease relative to 1st purge

Table S 2-4: Detected extractable impurities from **OEM,** **PVDF** membrane filter using Quadrupole-time-of flight coupled with ultra-high performance liquid chromatography.

Detected *m/z**	RT (min)	Tentative adducts	Molecular formulae	Mass error (ppm)	Signals intensities			Kruskal-Wallis (*p*-value)[a]	Fold-changes [b]
					1st purge	3rd purge	Reduction of impurities, %		
274.27292	5.5	$[M+H]^+$	$C_{16}H_{35}NO_2$	4.16	7726	998	87.08	●●●	-1.46
288.28856	5.7	$[M+NH_4]^+$	$C_{17}H_{34}O_2$	4.24	45422	4070	91.04	●●●	-1.94
301.13964	6.0	$[M+Na]^+$	$C_{16}H_{22}O_4$	4.99	11150	381	96.58	●	-0.79
205.08491	6.1	$[M+H]^+$	$C_{12}H_{12}O_3$	4.95	5050	158	96.87	●	-0.85
316.31927	6.3	$[M+NH_4]^+$	$C_{19}H_{38}O_2$	5.82	7525	997	86.75	●●●	-1.97
353.26433	6.9	$[M+Na]^+$	$C_{19}H_{38}O_4$	5.75	75362	1289	98.29	●●●	-1.64
313.27203	6.9	$[M+H]^+$	$C_{19}H_{36}O_3$	5.42	16267	100	99.39	●●●	-1.85
331.28313	7.1	$[M+H]^+$	$C_{19}H_{38}O_4$	3.50	7477	N/A	100.00	-	-
381.29515	7.5	$[M+Na]^+$	$C_{21}H_{42}O_4$	6.64	58864	716	98.78	●●	-1.61
341.30298	7.5	$[M+H]^+$	$C_{21}H_{40}O_3$	6.00	9670	N/A	100.00	-	-
359.31373	7.5	$[M+H]^+$	$C_{21}H_{42}O_4$	5.18	6045	N/A	100.00	-	-
492.40173	8.1	$[M+NH_4]^+$	$C_{30}H_{50}O_4$	6.34	79756	432	99.46	●●●	-6.47
520.43296	8.7	$[M+NH_4]^+$	$C_{32}H_{54}O_4$	6.12	169811	632	99.63	●●●	-6.91
534.44833	8.9	$[M+NH_4]^+$	$C_{33}H_{56}O_4$	6.50	5171	143	97.23	●●●	-5.43
663.44876	9.7	$[M+H]^+$	$C_{38}H_{62}O_9$	-3.17	32161	1446	95.50	●●	-1.87
721.50163	9.7	$[M+H]^+$	$C_{38}H_{73}O_{10}P$	-0.30	74355	5246	92.94	●●●	-1.92
701.40461	9.7	$[M+Na]^+$	$C_{34}H_{62}O_{13}$	5.38	6687	852	87.26	●●●	-1.39
685.43059	9.7	$[M+Na]^+$	$C_{38}H_{62}O_9$	-2.99	8480	622	92.67	●●●	-2.71
526.51643	9.8	$[M+H]^+$	$C_{33}H_{67}NO_3$	5.60	5306	252	95.25	●●●	-3.62

* Listed molecules are single-charged

[a]●, *p*-value < 0.05; ●●, *p*-value < 0.01; ●●●, *p*-value < 0.001 determined by parametric Kruskal-Wallis test

[b]Fold changes of peak area relative to mean control. Log_2 transformed. Values > 0 represent increase relative to 1st purge and values < 0 represent decrease relative to 1st purge
